# Occupational exposure to ionizing radiation and risk of lymphoma subtypes: results of the Epilymph European case-control study

**DOI:** 10.1186/s12940-020-00596-9

**Published:** 2020-04-25

**Authors:** Giannina Satta, Matteo Loi, Nickolaus Becker, Yolanda Benavente, Silvia De Sanjose, Lenka Foretova, Anthony Staines, Marc Maynadie, Alexandra Nieters, Federico Meloni, Ilaria Pilia, Marcello Campagna, Marco Pau, Lydia B. Zablotska, Pierluigi Cocco

**Affiliations:** 1 Department of Medical Sciences and Public Health, University of Cagliari, SS554, km 4.500, 09042 Monserrato (Cagliari), Italy; 2 German Cancer Research Center, Heidelberg, Germany; 3 Catalan Institute of Oncology, Barcelona, Spain; 4 Department of Cancer Epidemiology and Genetics, Masaryk Memorial Cancer Institute and MF MU, Brno, Czech Republic; 5 Dublin City University, Dublin, Ireland; 6 Dijon University Hospital, Dijon, France; 7 Medical Center, University of Freiburg, Freiburg, Germany; 8 Department of Epidemiology and Biostatistics, School of Medicine, University of California-San Francisco, San Francisco, CA USA

**Keywords:** Ionizing radiation, Lymphoma, Diffuse large B cell lymphoma, Occupational exposure

## Abstract

**Background:**

Evidence linking risk of lymphoma and B-cell lymphoma subtypes to ionizing radiation is inconclusive, particularly at low exposure levels.

**Methods:**

We investigated risk of lymphoma (all subtypes), B-cell lymphomas, and its major subtypes, associated with low-level occupational exposure to ionizing radiation, in 2346 lymphoma cases and 2463 controls, who participated in the multicenter EpiLymph case-control study. We developed a job-exposure matrix to estimate exposure to ionizing radiation, distinguishing between internal and external radiation, and we applied it to the lifetime occupational history of study subjects, We calculated the Odds Ratio (OR) and its 95% confidence interval (95% CI) for lymphoma (all subtypes combined), B-cell lymphoma, and its major subtypes using unconditional, polytomous logistic regression adjusting for age, gender, and education.

**Results:**

We did not observe an association between exposure metrics of external and internal radiation and risk of lymphoma (all subtypes), nor with B-cell lymphoma, or its major subtypes, at the levels regularly experienced in occupational settings. An elevated risk of diffuse large B cell lymphoma was observed among the most likely exposed study subjects with relatively higher exposure intensity, which would be worth further investigation.

**Conclusions:**

Further investigation is warranted on risk of B cell lymphoma subtypes associated with low-level occupational exposure to external ionizing radiation, and to clarify whether lymphoma should be included among the cancer outcomes related to ionizing radiation.

## Introduction

The carcinogenic effects of ionizing radiation have been extensively studied, and the association between human exposure to high doses of ionizing radiation and risk of solid tumours and leukemia is well-characterized [1]. As it concerns hemolymphopoietic malignancies other than leukemia, results are conflicting. Some studies reported an increased risk of non Hodgkin Lymphoma (NHL) among subjects with long-term repeated low-dose occupational exposure to *x*-rays and γ-rays [2, 3], contrasting previous reports [4, 5]. Results of cohort studies of radiologists and radiology technicians were also inconsistent [6, 7]. Instead, the Japanese A-bomb survivors, and Chernobyl cleanup workers had a slight excess risk of NHL and a significant excess of chronic lymphocytic leukemia (CLL) [8–11]. A previous population based case-control study explored NHL risk in relation to occupational exposure to ionizing radiation first with a standard job-exposure matrix (JEM) [12], and afterwards using a local JEM created for the purpose [13]; in both instances, the authors did not observe an association. Another population-based case-control study was conducted in Canada, which grouped the occupations by binary categorization of several occupational exposures, including radium, and uranium [14]: the results showed a 2- to 3-fold excess risk of NHL associated with occupations possibly involving such exposures, covering the broad spectrum of ionizing radiation, from α to *γ* radiation. As it concerns exposure to diagnostic x-ray procedures, risk of chronic lymphocytic leukemia (CLL), and non-Hodgkin Lymphoma (NHL) did not show an association, while risk of multiple myeloma (MM) increased with increasing number of diagnostic x-ray procedures [15]. A recent study of exposure to diagnostic radiation, based on the same data set we used for the present analysis, also did not corroborate the hypothesis of an increased risk of lymphoma overall nor of any of its major subtypes (Pasqual E et al. submitted). On the other hand, NHL incidence increased with latency among patients who underwent radiotherapy for non-small cell lung cancer [16], and for ankylosing spondylitis [17].

A link between exposure to ionizing radiation and lymphoma is plausible, as lymphocytes are highly radiosensitive; in fact, late damaging effects may show up even after years of exposure to external ionizing radiation [8, 18].

The study of cancer risk associated with exposure to low-level ionizing radiation has attracted a great deal of interest to establish whether a dose-response curve keeps being visible at the current occupational standards [19, 20]. Besides, the case-control studies conducted thus far did not distinguish whether internal or external doses of ionizing radiation were considered, which could have mislead the interpretation of findings [12–14]. It has been suggested that future epidemiological investigations should carefully address such interpretative limitations [21].

The aim of our study was to explore the association between occupational exposure to ionizing radiation and major lymphoma subtypes, separately for external or internal radiation, in a population-based European multicenter case-control study.

## Materials and methods

### Study population

The EpiLymph study is a multicenter case-control study on lymphoma etiology, which was conducted in 1998–2004 in six European Countries, namely Czech Republic, France, Germany, Ireland, Italy, and Spain. A detailed description of the study can be found elsewhere [22]. Briefly, eligible cases were all consecutive adult patients first diagnosed with lymphoma during the study period, resident in the referral area of the participating centers. The diagnosis was classified according to the 2001 WHO classification of lymphoma [23]; slides of about 20% of cases from each centre were reviewed centrally by a team of pathologists. Controls from Germany and Italy were randomly selected from the general population, frequency matched to cases on gender, 5-year age-group, and residence area. In the other countries, hospital controls with a diagnosis other than cancer, infectious diseases, and immunodeficiency were recruited. Overall, the data base included 2362 cases and 2465 controls who participated in the study. We excluded 14 cases as the diagnosis was not confirmed, and three controls because of missing work history. We also reclassified one case as a control, and excluded another case because of missing data on core variables, which resulted in 2346 cases and 2463 controls.

Local Ethics Committees approved the study protocol in all the participating centers. Informed consent was obtained from each participant. Overall, the participation rate was 88% in cases, 81% in hospital controls and 52% in population controls. Trained interviewers conducted in-person interviews with cases and controls, using the same standardized questionnaire translated to the local language. Questionnaire information included socio-demographics, lifestyle habits, such as alcohol drinking and tobacco smoking, health history, and a lifetime occupational history. For each job entry in the occupational history lasting 1 year or longer, the questionnaire included a short description of the type of business or trade; the specific individual tasks, and the tools used were also inquired into. Finally, the interviewer submitted to the study participant a checklist of risk factors of a priori interest for self-report about exposure. In case of occurrence of one or more exposure of interest, the interviewer also submitted one or more of fourteen specific job modules to gather additional details on the exposure circumstances. When planning the study, however, ionizing radiation was not included among the occupational exposures of interest.

### Occupational exposure assessment

Industrial hygienists in each participating center coded the work histories of study subjects using the 5-digit 1968 International Labour Organization Standard Classification of Occupations (ISCO68) [24], and the 4-digit codes of the 1996 European Statistical Classification of Economic Activities, revision 1 (NACE96) [25]. To assess occupational exposure to ionizing radiation, distinguishing between external and internal radiation, we developed a job-exposure matrix (JEM) based upon the list of ISCO68 and NACE96 codes. Exposure was classified according to the following exposure indicators:

- Dose of exposure to external and internal ionizing radiation by job was abstracted from published reports and surveillance data (see supplementary list of references), and unpublished data bases of monitoring data. For internal radiation, we first identified the main radionucleide conveying exposure in each job; secondly, to calculate the committed effective dose (CED) for adults associated with each radionucleide, we estimated the appropriate quality factor (QF) based upon the respective proportion of the different type of radiation emitted (QF = 20 for α radiation; QF = 1 for β, γ and *x* radiation; QF = 5 for protons and neutrons; a QF = 5 was assumed for neutrons in absence of specific information on their energy level) [4]; thirdly we applied the QF and the tissue weighting factor for circulating blood cells, as indicated in the BEIR VI report for radon [26]. Exposure was then categorized into four categories: unexposed, low, medium, and high. As a cut-off for exposure, we used the thresholds for the general population, i.e. 1 mSv/year for exposure to external radiation, or 20 mSv/year as a CED to the blood cells for internal exposure to α-radiation, correspondent to a dose equivalent of 1 mSv for radon [27]. A high exposure level was defined when available surveillance data suggested equivalent doses potentially equal to or above 6 mSv/year for external radiation, or to 44 mSv as a CED to the blood cells, correspondent to the 100 Bq/m^3^ (average in one year) external radiation level for radon at which lung cancer risk significantly increases, as indicated at the point 22 of the preamble of the EU Euratom Directive 2013/59 [28]. The cut point between the low and medium dose levels was arbitrarily set at 2.5 mSv, or 30 mSv-year as a CED to the blood cells for internal exposure to α-radiation. Jobs considered as benchmarks for each level were used as reference for occupations for whom no surveillance data were available.
- Probability of exposure: this metric was based on the proportion of the exposed within the occupational or the industrial code, with the following cut points: less than 20%, 21–80%, and more than 80%. Based on such cut points, we categorized probability of exposure into four categories: unexposed, possible, probable, and definite,

The dose and probability estimates associated with some occupational and industry codes were adapted in consideration of time and spatial changes in regulatory legislation and technology:

1. The probability estimate for power plant operators (ISCO68 code 96100) was differentiated by country, as a function of the proportion of energy from nuclear power plants [29]:

- high probability: France (100% of electric power from nuclear fuel);
- medium probability: Germany, Spain, Czech republic (30–50% of electric power from nuclear fuel);
- unexposed: Italy, Ireland (0% of electric power from nuclear fuel).

2. The estimate of potential dose in medical *x*-ray technicians (ISCO68 codes 07700 and 07710), and other medical, dental, veterinary and related workers (ISCO68 code 07990), was modulated by years of holding the job, considering the progressive reduction in exposure as a result of regulatory legislation over the time [30, 31]:

- high doses, if job started before 1975;
- medium doses, if job was initiated between 1975 and 1984;
- low doses, if job started from 1985 onwards.

3. Both, the dose and probability estimates for protective service workers, not elsewhere classified (ISCO68 code 58900), and mail sorting clerks (ISCO68 code 37020) was modulated by the approximate year of initiating use of *x*-rays in screening baggage, personal belongings, and mail:

- no exposure, if job started before 2002 in post office clerks, and before 1995 in airport security officers;
- low dose and probability, if job started from 2002 onwards for post office clerks, and from 1995 onwards for airport security officers. The low probability category was set as the proportion of airport security officers within the ISCO68 code 58900 was deemed to be lower than 20%. Measurements of external radiation doses in these occupations have shown levels ranging 52–1820 micro Roentgen (μR) per hour, corresponding to an annual dose up to 0.3 mSv, well below the threshold of 1 mSv/year [32], which explains why these workers are usually not considered as occupationally exposed, and thus are not required to wear a dosimeter. We decided nonetheless to include this additional occupational source, as it would add to the background radiation level.

Baggage control personnel in ship boarding was considered as unexposed, as the use of *x*-rays for screening baggage and personal belongings before allowing boarding onto ships was introduced when recruitment of study subjects was approximately completed.

The estimated dose and probability of exposure for each study subject was calculated as the time weighted average of the respective estimates for each job entry in his/her work history. The lifetime weighted average doses were finally categorized as described above. Cumulative exposure to external or internal ionizing radiation for each individual was obtained as the sum of the products of the dose estimate associated to each job entry times its duration in years. For the purposes of analysis, cumulative doses were categorized into tertiles based on the distribution among the controls.

Due to the small number of subjects in the high category of exposure to external and internal radiation, in conducting the analysis, we combined the medium and high probability categories, and the medium-high categories of average dose of exposure, and we used the median as the cut point between increasing cumulative exposure categories.

### Statistical methods

The Odds Ratio (OR) and its 95% confidence interval (CI) of lymphoma (all subtypes combined), B-cell lymphoma, and its most prevalent subtypes, namely diffuse large B cell lymphoma (DLBCL), CLL including small lymphocytic lymphoma (CLL/SLL), follicular lymphoma (FL), multiple myeloma (MM), and Hodgkin Lymphoma (HL), were calculated using unconditional logistic regression and polytomous logistic regression analysis. We conducted separate analyses for exposure to external and internal ionizing radiation; to single out the effects of the two different types of ionizing radiation, we excluded 70 subjects (27 cases and 43 controls) exposed to internal radiation from the unexposed to external radiation, and 410 subjects (190 cases and 220 controls) exposed to external radiation from the unexposed to internal radiation. We coded separately 18 subjects exposed to both types of radiation, so to inquiry into effects resulting from their sum. Therefore, we conducted the analysis for external radiation on 2319 cases and 2420 controls, and that for internal radiation on 2156 cases and 2243 controls. We looked at each exposure metric individually, i.e. we did not combine probability and dose of exposure in the same exposure metric, nor included them together in the same model. The rationale for such decision was to explore the association from two different perspectives, so to increase the strength of inference. All models included age, gender, education, and study center as the adjusting covariates. Heterogeneity in risk across lymphoma subtypes was assessed with the Cochrane Q test.

The test for linear trend across categories of the exposure metrics was calculated using the Wald statistics after continuous transformation of the covariates in the logistic model. We set the two-tailed α-error threshold to reject the null hypothesis at *p* < 5%. All the analyses were conducted with SPSS® version 20.0.

## Results

Mean age was substantially similar for cases and controls (cases: 56.1 years, *sd* 16.2;controls: 56.2 years, *sd* 16.0), and by gender (men: 56.0 years, *sd* 15.8; women: 56.3 years, *sd* 16.4).The male/female ratio among the cases was 1.3:1, consistent with the existing literature [33]. Table 1 shows the distribution of cases and controls by participating center and by selected variables.

**Table 1:** Distribution of cases and controls by gender, area of study, education level, and ever exposure to external and internal sources of ionizing radiation

	*Cases* *(N = 2346)*		*Controls* *(N = 2463)*	
	N	%	N	%
*Gender*				
Male	1313	55.97	1321	53.63
Females	1033	44.03	1142	46.37
*Area of recruitment*				
Spain	591	25.19	631	25.62
France	298	12.70	276	11.21
Germany	703	29.97	710	28.83
Italy	262	11.17	336	13.64
Ireland	201	8.57	207	8.40
Czech Republic	291	12.40	303	12.30
*Education level*				
Elementary/middle school	1078	45.95	1122	45.55
High school	937	39.94	1001	40.64
University degree	331	14.11	340	13.81
*Ever occupationally exposed to ionizing radiation*				
External radiation	224	9.66^a^	255	10.53^a^
Internal radiation	37	1.72^b^	54	2.23^b^
External and internal radiation	8	0.34^a^	10	0.41^a^

Notes: ^a^ Percentage over the 2319 cases and 2422 controls included in the analysis of external radiation

^b^ Percentage over the 2156 cases and 2245 controls included in the analysis of internal radiation

Having ever being occupationally exposed to external ionizing radiation was not associated with risk of lymphoma (all subtypes combined; OR = 0.9; 95% CI 0.73, 1.08); the result was likewise after restricting the analysis to B-cell lymphomas (OR = 0.9; 95% CI 0.75, 1.14) (Table 2a). Risk estimates did not increase by cumulative exposure, duration, or maximum or average intensity of exposure, nor did they increase after limiting the analysis to the categories of probable and definite exposure to cumulative doses above 15.4 mSv-years (lymphoma, any subtype: OR = 1.0, 95% CI 0.51, 2.06; based on 16 exposed cases and 16 exposed controls; B-cell lymphoma: OR = 1.0, 95% CI 0.48,2.12; based on 13 exposed cases and 16 exposed controls), with no upward trend detected (*p* for trend = 0.89 and 0.97, respectively). No association was observed between risk of lymphoma (all subtypes combined) or B-cell lymphomas and exposure to internal radiation (Table 2b). Note that numbers of cases and controls in Table 3a and b differ because of the exclusion of subjects exposed to internal radiation from the unexposed to external radiation (Table 2a), and of subjects exposed to external radiation from the exposed to internal radiation (Table 2b).

**Table 2:** OR and 95% CI for lymphoma (all subtypes combined) and B-cell lymphomas in relation to dose and probability of exposure to external (A) and internal (B) ionizing radiation

(A) external radiation and (B) interal radiation	*All lymphomas* *(N = 2319)*			*B-cell lymphomas* *(N = 1907)*		
	cases/controls	OR	95%CI	cases/controls	OR	95%CI
(A)						
Unexposed	2095/2164	1.0	–	1730/2164	1.0	–
Ever exposed	224 /255	0.9	0.73–1.08	177/255	0.9	0.75–1.14
*Average probability*						
Possible	199/228	0.9	0.73–1.08	156/228	0.9	0.74–1.15
Probable/definite	25/27	0.9	0.53–1.60	21/27	0.9	0.52–1.69
*Average dose*						
1–2.5 mSv	103/121	0,8	0.62–1.08	85/121	0.9	0.65–1.16
≥ 2.6 mSv	121/134	1.0	0.74–1.24	92/134	1.0	0.74–1.30
≥ 2.6 mSv, probable/ definite exposure	16/13	1.3	0.60–2.64	12/13	1.2	0.53–2.60
*Duration of exposure*						
≤ 11 years	116/131	0.9	0.68–1.15	85/131	0.9	0.68–1.21
≥ 12 years	108/124	0.9	0.68–1.17	92/124	0.9	0.71–1.25
*Cumulative exposure*						
1.75–15.39 mSv-years	119/126	0.9	0.72–1.21	90/126	1.0	0.72–1.27
> 15.4 mSv-years	105/129	0.9	0.65–1.11	87/129	0.9	0.68–1.19
Probable/definite exposure						
≥ 15.4 mSv-years	16/16	1.0	0.51–2.06	13/16	1.0	0.48–2.12
	*All lymphomas* *(N = 2186)*			*B-cell lymphomas* *(N = 1700)*		
	cases/controls	OR	95%CI	cases/controls	OR	95%CI
(B)						
Unexposed	2149/2164	1.0	–	1669/2164	1.0	–
Ever exposed	37/54	0.7	0.43–1.02	31/54	0.7	0.45–1.12
*Average probability*						
Possible	13/22	0.6	0.28–1.12	11/22	0.6	0.31–1.33
Probable/definite	24/32	0.7	0.39–1.11	20/32	0.8	0.43–1.34
*Average intensity*						
1–2.5 mSv	35/30	1.2	0.70–1.90	29/30	1.1	0.65–1.85
> 2.5 mSv	2/24	0.1	0.02–0.31	2/24	0.1	0.03–0.50
*Duration of exposure*						
≤ 8 years	24/27	0.9	0.49–1.50	19/27	0.9	0.50–1.65
≥ 9 years	13/27	0.5	0.24–0.91	12/27	0.5	0.26–1.04
*Cumulative exposure*						
≤ 30 mSv-years	21/25	0.8	0.45–1.46	18/25	0.9	0.50–1.71
> 30 mSv-years	16/29	0.5	0.29–1.00	13/29	0.5	0.27–1.03

**Table 3:** OR and 95% CI for lymphoma subtypes by intensity and confidence of exposure to external (A) and internal (B) ionizing radiation

	***DLBCL***			***Follicular lymphoma***			***CLL***			***Multiple Myeloma***			***Hodgkin Lymphoma***		
	***Cases/Controls***	***OR***	***95%CI***	***Cases/Controls***	***OR***	***95%CI***	***Cases/Controls***	***OR***	***95%CI***	***Cases/Controls***	***OR***	***95%CI***	***Cases/Controls***	***OR***	***95%CI***
(A)															
Unexposed	477/2164	1.0	–	219/2164	1.0	–	362/2164	1.0	–	260/2164	1.0	–	283/2164	1.0	–
Ever exposed	55/255	0.9	0.69–1.29	31/255	1.1	0.77–1.72	45/255	1.2	0.82–1.65	15/255	0.5	0.31–0.93	34/255	0.8	0.50–1.13
*Average probability*^a^															
Possible	44/228	0.9	0.61–1.21	27/228	1.1	0.72–1.71	41/228	1.2	0.85–2.76	14/228	0.7	0.32–0.99	30/228	0.7	0.47–1.11
Probable/ definite exposure	11/27	1.6	0.80–3.40	4/27	1.4	0.49–4.29	4/27	0.8	0.26–2.31	1/27	0.3	0.04–2.50	4/27	1.0	0.34–3.21
*Average intensity*^a^															
1–2.5 mSv	24/121	0.9	0.56–1.40	15/121	1.3	0.74–2.33	23/121	1.0	0.62–1.62	8/121	0.5	0.24–1.06	12/121	0.7	0.34–1.26
≥ 2.6 mSv	31/134	1.0	0.66–1.50	16/134	1.0	0.59–1.77	22/134	1.4	0.85–2.26	7/134	0.6	0.27–1.28	22/134	0.8	0.49–1.36
≥ 2.6 mSv probable/ definite exposure	7/13	2.3	0.90–5.84	2/13 0	1.4	.32–6.58	2/13	1.0	0.21–4.47	1/13	0.8	0.10–6.54	4/13	2.0	0.59–6.81
*Duration of exposure*^a^															
≤ 11 years	28/131	1.0	0.62–1.46	11/131	0.8	0.41–1.48	20/131	1.1	0.69–1.90	7/131	0.6	0.28–1.24	22/131	0.6	0.39–1.07
≥ 12 years	27/124	1.0	0.60–1.44	20/124	1.5	0.93–2.53	25/124	1.2	0.75–1.87	8/124	0.5	0.23–1.01	12/124	1.0	0.52–1.86
*Cumulative exposure*^a^															
1.75–15.39 mSv-years	27/126	1.0	0.55–1.81	15/126	0.5	0.15–1.53	21/126	1.1	0.57–2.18	8/126	0.7	0.27–1.74	20/126	0.5	0.20–1.06
≥ 15.4 mSv-years	28/129	0.9	0.62–1.28	16/129	1.3	0.82–1.97	24/129	1.2	0.82–2.82	7/129	0.5	0.26–0.98	14/129	0.8	0.51–1.31
Probable/definite exposure															
≥ 15.4 mSv-years	7/16	1.6	0.71–3.46	2/16	1.3	0.37–4.34	3/16	0.8	0.23–2.73	1/16	0.5	0.06–3.57	3/16	1.1	0.36–3.57
	***DLBCL***			***Follicular lymphoma***			***CLL***			***Multiple Myeloma***			***Hodgkin Lymphoma***		
	***Cases/Controls***	***OR***	***95%CI***	***Cases/Controls***	***OR***	***95%CI***	***Cases/Controls***	***OR***	***95%CI***	***Cases/Controls***	***OR***	***95%CI***	***Cases/Controls***	***OR***	***95%CI***
(B)															
Unexposed	476/2164	1.0		219/2164	1.0		362/2164	1.0		260/2164	1.0		304/2164	1.0	
Ever exposed	8/54	0.6	0.28–1.28	2/54	0.5	0.11–1.93	4/54	0.8	0.37–1.75	3/54	0.5	0.15–1.58	2/54	0.1	0.03–0.64
*Average intensity*															
1–2.5 mSv	7/22	1.0	0.45–2.40	2/22	0.7	0.17–3.09	4/22	1.1	0.49–2.47	2/22	0.5	0.12–2.09	2/22	0.8	0.17–3.78
≥ 2.5 mSv	1/32	0.1	0.02–1.07	0/32	–		0/32	–		1/32	0.5	0.06–3.58	0/32	–	
*Average probability*															
Possible	2/30	0.4	0.09–1.60	1/30	0.5	0.07–3.80	1/30	0.6	0.13–2.55	1/30	0.4	0.05–3.10	1/30	0.2	0.03–1.63
Probable/definite	6/24	0.8	0.31–1.83	1/24	0.4	0.06–3.15	3/24	0.9	0.37–2.32	2/24	0.5	0.12–2.26	1/24	0.1	0.01–0.89
*Duration of exposure*															
≤ 8 years	7/27	1.1	0.47–1.57	1/27	0.5	0.07–3.87	2/27	0.9	0.31–2.79	2/27	0.7	0.15–2.82	2/27	0.2	0.05–1.02
≥ 9 years	1/27	0.1	0.02–1.05	1/27	0.4	0.06–3.10	2/27	0.7	0.24–2.09	1/27	0.3	0.04–2.37	0/27	–	
*Cumulative exposure*															
30 mSv-years	5/25	0.9	0.32–2.29	1/25	0.5	0.07–3.73	2/25	1.0	0.33–2.94	2/25	0.7	0.16–3.05	2/25	0.3	0.06–1.23
30 mSv-years	3/29	0.4	0.12–1.31	1/29	0.4	0.06–3.19	2/29	0.7	0.23–2.01	1/29	0.3	0.04–2.20	0/29	–	

^a^Tests for trend

DLBCL: average probability = 0.144, *p* = 0.89; duration of exposure = −0.366, *p* = 0.71; average intensity with probable/definite exposure = 1.673, *p* = 0.09; cumulative exposure with probable/definite exposure = 1.284; *p* = 0.20

FL: average probability = 0.768, *p* = 0.44; duration of exposure = 1.218, *p* = 0.22; average intensity with probable/definite exposure = 0.597, *p* = 0.55; cumulative exposure with probable/definite exposure = 1.075; *p* = 0.28

HL test for trend average intensity with probable/definite exposure = 0.595, *p* = 0.55

Table 3 shows the results by lymphoma subtype. The number of the exposed was very small for each subtype, particularly in the high dose and high probability categories of exposure. None of the lymphoma subtypes we analyzed showed an association with ever exposure to external ionizing radiation. For external radiation, the statistical power was not enough to detect as significant the increase in risk of DLBCL associated with probable/definite exposure (OR = 1.6, 95% CI 0.80, 3.40), probable/definite exposure to estimated average doses ≥2.6 mSv (OR = 2.3; 95% CI 0.90, 5.84; based on seven cases and 13 controls), and probable/definite exposure to cumulative doses above 15.5 mSv-years (OR = 1.6; 95% CI 0.71, 3.46; based on seven cases and 16 controls), with no trend detected by both exposure metrics (*p* = 0.09, and *p* = 0.16, respectively). Chance most likely explained the moderate increase in the risk estimate for follicular lymphoma associated with duration of exposure above the median among subjects with probable/definite exposure (OR = 2.4, 95% CI 0.76, 7.41), probable/definite exposure to average doses ≥2.6 mSv (OR = 1.4, 95% CI 0.32, 6.58), and cumulative doses ≥15.4 mSv-years (OR = 1.6, 95% CI 0.71, 3.46), with no upward trend detected (*p* = 0.28, *p* = 0.55, and *p* = 0.28, respectively). A sporadic excess risk of Hodgkin lymphoma associated with probable/definite exposure to average doses above 2.6 mSv (OR = 2.0, 95% CI 0.59, 6.81) was also interpreted as a chance finding.

Internal doses from occupational exposure did not show an association with risk of any of the major lymphoma subtypes (Table 3b). For all the subtypes, risks associated with the exposure metrics were below or around unity in all cells, with no heterogeneity detected (Q = 4.31, *p* = 0.366). An analysis restricted to subjects exposed to both internal and external radiation also did not show an association (not shown in the Tables).

## Discussion

According to our assessment, in our population based case-control study, 10.1% study subjects were ever exposed to external ionizing radiation, but only for 1.2% exposure was probable or definite. The proportion of study subjects exposed to internal ionizing radiation was even lower. Under such circumstances, we could only have enough statistical power to detect risks of lymphoma (all subtypes combined) of 2.05 or greater. Therefore, in spite of its large size, our study was underpowered to provide evidence of an association between low-level occupational exposure to external or internal ionizing radiation and risk of lymphoma subtypes. The increasing risk of DLBCL, observed in with different exposure metrics among subjects with probable/definite exposure would warrant further investigation before discarding it as a chance finding. We did not find evidence of an association between exposure to internal radiation, which, in occupational settings, is mostly due to inhalation of α-radiation, and risk of any of the lymphoma subtypes most frequently represented among our cases.

Previous cohort studies found an increase in NHL risk following exposure to external ionizing radiation in the aftermath of the Hiroshima and Nagasaki bombings [6, 12, 33] or following radiotherapy [3]. On the other hand, findings were contradictory when occupational exposures were considered [5, 8]. HL incidence and mortality, and NHL incidence were associated with cumulative dose of external radiation among workers at the Springsfield uranium production facility [34]. Also, mortality from lymphatic and hematopoietic malignancies other than leukemia was elevated in a cohort study of seven Colorado uranium mills (SMR = 1.44; 95% CI 0.83–2.35), due to excess cases of lymphoma, Hodgkin and non-Hodgkin [35]. Risk of CLL, HL, and NHL, were also elevated in other cohort studies of uranium and non uranium miners in relation to exposure to γ radiation [36, 37], and in recent studies of Chernobyl cleanup workers with prolonged exposure to low-dose external ionizing radiation [13, 14], while MM results were conflicting [13, 14, 38, 39]. However, studies of uranium miners and millers are difficult to interpret because of the small cohort size, the lack of measurements or estimates of internal doses, and by the use of surrogates for cumulative exposure to uranium. In fact, unavailability of historical monitoring data reflects a common drawback of many retrospective studies, including those conducted in uranium miners and millers [39]. Some studies also reported a link with internal exposure resulting from inhalation of radon gas and its rapidly decaying α-emitting daughters, exhaling from the porosity of the rocks, and not diluted by an efficient ventilation system [36, 39]. Previous large surveys contradict such reports [7, 8]. Besides, the International Agency of Research on Cancer classified uranium in group 3 (not classifiable), and it did not include NHL or CLL among the neoplastic diseases induced by exposure to α radiation [38]. Apart from a few exceptions, previous studies considered all lymphomas as a unique disease entity, or at most made a distinction between Hodgkin and non-Hodgkin lymphomas. However, recent collaborative studies from the InterLymph Consortium have shown etiological heterogeneity across lymphomas in terms of occupational risk factors, lifestyle habits, health history, and individual characteristics [40]. Therefore, if only a few lymphoma subtypes were radiosensitive, dilution of the association with the relevant outcome might explain previous contradictory findings.

Besides the generic definition of the disease entity, random fluctuation of the risk estimates and/or exposure misclassification might explain the lack of a consistent link between exposure to ionizing radiation and lymphoma. Using a JEM to retrospectively assess occupational exposures in population-based studies conveys a certain degree of exposure misclassification in respect to the gold standard of available monitoring data. A reduction in sensitivity is expected as a JEM might not cover the whole spectrum of jobs implying possible penetration of ionizing radiation into the organisms through different routes; specificity might be even more reduced as the job codes, and not the individual measurements, are the units to classify exposure. We tried to minimize the consequences of exposure misclassification, by designing our JEM a priori on the list of the ISCO68 and the NACE96 codes, and by applying it subsequently to the consistently coded work histories of study subjects. Any misclassification affecting our findings, therefore, might have been non differential between cases and controls, and its effect would have been towards a dilution of the true association and an underestimate of the true risk, if any. Also, we refrained from lumping together different exposure metrics, such as intensity and probability, as we endorse the opinion that such strategies would result in both systematic and differential measurement errors [41].

Other JEMs are available for the assessment of exposure to ionizing radiation, such as the job-exposure matrix for exposure to carcinogens initially developed by the Finnish Institute of Occupational Health (FINJEM). FINJEM is a development of the EU CAREX (CARcinogen EXposure) system, the job-exposure matrix for exposure to carcinogens initially developed by the Finnish Institute of Occupational Health with the support from the International Agency for Research on Cancer, and subsequently adapted to studies in numerous world countries [42, 43]. However, FINJEM classifies exposure to radon decay products, but not α radiation overall. The Canadian version of CAREX, also widely used, does not discriminate between types of radiation, and both FINJEM and CAREX are based on occupational codes only, which could lead to some loss of information, difficult to quantify. For these reasons, we did not rely on the existing JEMs, and developed instead our own JEM. An attempt to combine the assessments from the various existing JEMs to come up with more robust estimates will be pursued in the near future.

A further limitation in interpreting our findings is related to the multiple comparisons we made, which generated the random fluctuations of the risk estimates we observed in some subsets.

Apart from chance, we do not have a clear explanation of the inverse association between risk of lymphoma and its subtypes and the exposure metrics of internal radiation doses. Several limitations in our study might have played a role. First, the conditions inside workplaces may vary, and they may affect the equilibrium, for instance between radon and its decay products, and therefore the exposure. However, we did not have the necessary information to include such source of variation in our estimated by occupation and industry. Second, the size of particles engulfing the α-emitting radioisotope, typically radon and its decay products, may vary between underground and open air mines and quarries, with smaller particles more likely to penetrate the deep lung and to deliver larger doses to the lungs and the other internal organs. Particle size would be smaller in mines; however, lymphoma risk associated with underground mining in our study was also reversed (OR = 0.7; 95% CI 0.55, 0.96; not shown in the Tables), which would undermine an effect of particle size in masking an association with internal radiation exposure in our study.

Third, in the general population, exposure during diagnostic procedures is a major source of external dose of ionizing radiation, which we did not consider in our paper. Another paper used the same data base as in our study to investigate the association between cumulative doses of ionizing radiation from diagnostic procedures in relation to risk of lymphoma (Pasqual E et al. submitted): the median cumulative dose at the bone marrow level was 2.25 mGy, much below the estimated median cumulative dose among the exposed to external ionizing radiation in our study, and there was no association with risk of lymphoma. Also, there is no plausible reason to suspect that cases in our study had less radiodiagnostic procedures in respect to the controls, It seems therefore unlikely that exposure from diagnostic procedures might have acted as a confounder in our study.

## Conclusions

In conclusion, our results did not show an association of lymphoma risk with low level occupational exposure to ionizing radiation, either external or internal, within the current standards. Occupational exposure to radiation as a possible cause of lymphoma subtypes warrants further investigation with larger data sets. Pooled analyses of large case-control studies would be particularly suitable to test whether the range of haemopoietic malignancies related to exposure to ionizing radiation should be extended to cover also specific lymphoma subtypes.

## Supplementary information

**Additional file 1.** List of reference publications used in the exposure assessment.

## Data Availability

The EpiLymph dataset is publicly available upon reasonable request to the InterLymph Consortium Data Coordinating Center (Mayo Clinic, Rochester, MN; e-mail: Norman.Aaron@mayo.edu), with permission of the EpiLymph Steering Committee. The data generated and/or analysed during the current study are available upon reasonable request to the corresponding author, pending approval from the EpiLymph Steering Committee.

## Abbreviations

CI: 95% confidence interval
CLL: Chronic lymphocytic leukemia
DLBCL: Diffuse large B cell lymphoma
FL: Follicular lymphoma
HL: Hodgkin Lymphoma
ISCO68: 1968 Office International Labour International Standard Classification of Occupations
JEM: Job-exposure matrix
MM: Multiple myeloma
NACE96: 1996 European Statistical Classification of Economic Activities, revision 1
NHL: Non Hodgkin Lymphoma
OR: Odds Ratio
