# Supplementary material for: Occupational exposure to ionizing radiation and risk of lymphoma subtypes: results of the Epilymph European case-control study
Source: Environ Health. 2020 Apr 25;19:43. doi: 10.1186/s12940-020-00596-9 (PMC7183712; doi:10.1186/s12940-020-00596-9)
Supplement: Supplementary file 1 — Additional file 1. List of reference publications used in the exposure assessment. [file 12940_2020_596_MOESM1_ESM.docx]

Supplementary material

List of reference publications used in the exposure assessment

1. Council Directive 2013/59/Euratom of 5 December 2013: laying down basic safety standards for protection against the dangers arising from exposure to ionising radiation, and repealing Directives 89/618/Euratom, 90/641/Euratom, 96/29/Euratom, 97/43/Euratom and 2003/122/Euratom with Annexes.
2. Occupational Safety and Health Administration (OSHA), Department of Labour. Occupational Exposure to Ionizing Radiation, Washington, DC. Fed Register: May 3, 2005. Volume 70:22828-35; 2005.
3. Eurostat, Nuclear energy statistics, 1998-2004. <http://ec.europa.eu/eurostat/en/>. Accessed 12 November 2016.
4. Jones CG. A review of the history of U.S. radiation protection regulations, recommendations, and standards. Health Physics. 2005;88:105-24.
5. United Nations Scientific Committee on the Effects of Atomic Radiation (UNSCEAR). *2000 report to the General Assembly, with scientific annexes. Volume 1: Sources*. New York: United Nations Scientific Committee on the Effects of Atomic Radiation; 2000.
6. International Commission on Radiological Protection. ‘*The 2007 Recommendations of the International Commission on Radiological Protection*’ Annals of the ICRP, Volume 37/2-4; ICRP Publication 103 2008.
7. Committee on Health Risks of Exposure to Radon (BEIR VI), Commission on Life Sciences, National Research Council. National Research Council. Health effects of exposure to radon. BEIR VI. Washington, DC: National Academy Press, 1999.
8. Kathren RL. Historical Development of the Linear Nonthreshold Dose- Response Model as Applied to Radiation, 1 PIERCE L. REV. 5; 2002.
9. Archer VE. Occupational exposure to radiation as a cancer hazard. Cancer. 1977;39:1802–6.
10. Linet MS, Kim KP, Miller DL, Kleinerman RA, Simon SL, Berrington de Gonzalez A. Historical review of occupational exposures and cancer risks in medical radiation workers. Radiat Res. 2010;174:793-808.
11. Pierce DA, Shimizu Y, Preston DL, Vaeth M, Mabuchi K. Studies of the mortality of atomic bomb survivors. Report 12, Part I. Cancer: 1950-1990. Radiat Res. 1996;146:1-27.
12. Preston DL, Kusumi S, Tomonaga M, Izumi S, Ron E, Kuramoto A, et al. Cancer incidence in atomic bomb survivors. Part III. Leukemia, lymphoma and multiple myeloma, 1950-1987. Radiat Res. 1994;137/Suppl 2:S68-97.
13. Zenz C, Dickerson OB, Horvath EP. Occupational Medicine. St Louis, MO: Mosby publisher,1997.
14. International Agency for Research on Cancer. IARC Monographs on the Evaluation of Carcinogenic Risks to Humans. Ionizing Radiation, Part 1: X- and Gamma (g)-Radiation and Neutrons. Vol. 75. Lyon: IARC, 2000.
15. International Agency for Research on Cancer. IARC Monographs on the Evaluation of Carcinogenic Risks to Humans. Ionizing Radiation, Part 2: Some Internally Deposited Radionucleides. Vol. 78. Lyon: IARC, 2001.
